# Enriched and Sustained Oxygen Vacancies in Amorphous NiWO_x_ Enhance and Stabilize Urea Electrooxidation

**DOI:** 10.1002/anie.2821661

**Published:** 2026-07-08

**Authors:** Yinuo Wang, Ke Zhang, Mingxing Zhou, Shijin Zhang, Ling Zhao, Gongjin Chen, Luca Magagnin, Lu Wang, Yian Wang, Minhua Shao

**Affiliations:** ^1^ School of Civil Engineering, Key Laboratory of Water Supply & Sewage Engineering of Ministry of Housing and Urban‐rural Development Chang’an University Xi’an China; ^2^ Department of Chemical and Biological Engineering The Hong Kong University of Science and Technology Clear Water Bay Kowloon Hong Kong China; ^3^ Dipartimento di Chimica Materiali e Ingegneria Chimica Giulio Natta Politecnico di Milano Milan Italy; ^4^ Dongguan Key Laboratory of Artificial Intelligence Design for Advanced Materials School of Physical Sciences Great Bay University Dongguan China; ^5^ CIAC‐HKUST Joint Laboratory for Hydrogen Energy Energy Institute The Hong Kong University of Science and Technology Kowloon Hong Kong China; ^6^ Guangzhou Key Laboratory of Electrochemical Energy Storage Technologies Fok Ying Tung Research Institute The Hong Kong University of Science and Technology Guangzhou China

**Keywords:** amorphous structure, oxygen vacancy, urea oxidation reaction, nickel tungsten oxides, hydrogen production

## Abstract

Urea electrolysis is promising for energy‐saving hydrogen production and effective treatment of urea‐polluted water. However, the activity and stability of Ni‐based electrocatalysts for the anodic urea oxidation reaction (UOR) are limited by the lack of active NiOOH and strong intermediates adsorption. Although oxygen vacancies (O_v_) benefit for the UOR, continuous generation and stabilization of O_v_ remain challenges. Herein, we propose an “amorphous oxygen mechanism (AOM)” for urea electrooxidation in amorphous nickel tungsten oxide (NiWO_x_) with tunable O_v_ concentrations. Systematic experimental and theoretical studies demonstrate that enriched O_v,_ not only facilitate the formation of active NiOOH species, but also significantly reduce the energy barrier of the rate‐determining step. More importantly, the amorphous state allows more defects, which enables the in situ regeneration and sustainability of O_v_ during the UOR via a continuous oxygen escape in the amorphous catalyst. Notably, NiWO_x_ with the highest O_v_ achieves an ultralow potential of 1.34 V at 10 mA cm^−2^ with incredible 400 h stability. Moreover, only 1.46 V is demanded for urea electrolysis with 100 mA cm^−2^ in an anion‐exchange membrane electrolyzer. The long‐term stability is also impressive. This work highlights the significant role of amorphous structure, providing valuable insights into catalyst design in electrocatalysis.

## Graphical abstract

We propose an “amorphous oxygen mechanism (AOM)” for urea electrooxidation on amorphous NiWO_x_ with tunable O_v_, which achieves robust UOR activity and stability simultaneously. The “amorphous oxygen” in NiWO_x_ prefers to escape during the UOR, in situ generating and stabilizing O_v_, which thermodynamically promotes the reaction. These findings highlight the crucial role of the amorphous structure in the in situ generation and sustainability of O_v_.

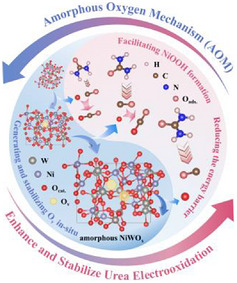

## 1 Introduction

Urea electrolysis (CO(NH_2_)_2_ + H_2_O → 3H_2_ + N_2_ + CO_2_) has garnered significant attention as an innovative technology that enables simultaneous wastewater purification and clean H_2_ production through the electrochemical conversion of urea [1–3]. Importantly, the thermodynamic equilibrium potential for hydrogen production via urea electrolysis is only 0.37 V versus the reversible hydrogen electrode (vs. RHE), significantly lower than 1.23 V required for conventional water electrolysis [4, 5]. This reduction in energy consumption offers substantial economic and environmental benefits, positioning urea electrolysis as a promising approach to synergize clean H_2_ and environmental remediation.

However, as an indispensable half‐reaction in the urea electrolysis system, the electrochemical urea oxidation reaction (UOR) involves a complex six‐electron transfer process with multiple intermediate adsorption and desorption steps [6, 7], resulting in sluggish reaction kinetics and high overpotential, which restrict overall energy efficiency. Therefore, developing high‐performance electrocatalysts is essential for practical application of the UOR. Ni‐based catalysts have been demonstrated the superior UOR performance compared to noble metals in alkaline media [8, 9, 10], primarily due to the electrochemically formed highly active NiOOH species [11, 12]. Thus, enhancing the generation of NiOOH is critical for improving the UOR performance. Theoretical studies have revealed that the highest energy barrier in the adsorbate evolution mechanism (AEM) pathway on NiOOH is the desorption of the *COO intermediate [13, 14, 15], which thermodynamically inhibits the UOR activity. Further investigations have demonstrated that the lattice oxygen in the crystalline catalysts can be activated and involved in the final desorption step due to weak M─O bonds at the surface, optimizing the energy state and favoring the UOR process [13, 16, 17]. This mechanism is referred to as the lattice oxygen mechanism (LOM) [18, 19]. Although LOM significantly promotes the UOR activity, the lattice oxygen escaping from the crystal triggers catalyst structural collapse and degradation of catalyst performance [20, 21, 22]. Therefore, development of the catalysts with robust activity as well as stability for the UOR remains to be challenging.

Defect engineering, especially oxygen vacancies (O_v_), plays a crucial role in modulating the physical and chemical properties of materials [23, 24, 25]. The introduction of O_v_ can provide abundant undercoordinated atoms as active sites and optimize the electronic structure of the catalysts, enhancing adsorption behavior toward key intermediates [26, 27]. However, O_v_ in crystalline catalysts often suffer from high surface energy and internal stress, which makes their promoting effect unsustainable [13, 20]. Instead, amorphous structure features the unique atomic arrangements of long‐range disorder and short‐range order [28, 29], facilitating the formation, stabilization, and migration of O_v_ [30]. Furthermore, amorphous catalysts offer greater flexibility for dynamic reconstruction of active sites compared to crystalline catalysts. Thus, the high concentration of defects in amorphous structures may allow sustainable oxygen escaping and create O_v_ continuously. A new mechanism with O_v_ in amorphous oxide catalysts would achieve both high activity and stability of the UOR.

In this study, we propose a novel “amorphous oxygen mechanism (AOM)” for the first time, owning to a more thermodynamically and kinetically favorable UOR by generating enriched and sustained O_v_ in our established amorphous NiWO_x_ framework. The amorphous NiWO_x_ (x < 4) with tunable O_v_ concentration were synthesized via a facile air annealing strategy at various temperatures, establishing a platform to systematically investigate the correlation between the O_v_ content and the UOR performance. Physical and electrochemical characterizations demonstrate that, a higher O_v_ content in amorphous NiWO_x_ enhances the formation of NiOOH species and reduces the activation energy for the UOR. Combined with density functional theory (DFT) calculations, it is noted that the increased O_v_ content significantly lowers the energy barrier and facilitates the escape of oxygen from the surface, sustainably generating more O_v_ during the UOR process, resulting in an “amorphous oxygen mechanism (AOM)”. The proposed AOM, building upon the LOM, involves oxygen atoms from the amorphous catalyst participating directly in the reaction, which confirmed by time‐of‐flight secondary ion mass spectrometry (ToF‐SIMS) and in situ differential electrochemical mass spectrometry (DEMS), thereby reducing the energy barrier and accelerating UOR. Moreover, the amorphous structure in AOM can better stabilize the O_v_ formed after oxygen departure, mitigating the structural instability often associated with conventional LOM and producing sustainable O_v_ in the catalyst. With the assistance of enriched and sustained O_v_ in amorphous NiWO_x_ catalysts, high activity and stability are simultaneously achieved in UOR. Owing to the inherent structural flexibility of amorphous materials, this work underlines their significant potential in electrocatalysis, particularly as a suitable host for sustaining high concentrations of O_v_. These findings provide a catalyst design principle for developing efficient electrocatalysts through O_v_ engineering.

## 2 Results and Discussion

### 2.1 Synthesis and Characterization of NiWO_x_ with Tunable O_v_ Concentrations

**FIGURE 1:**
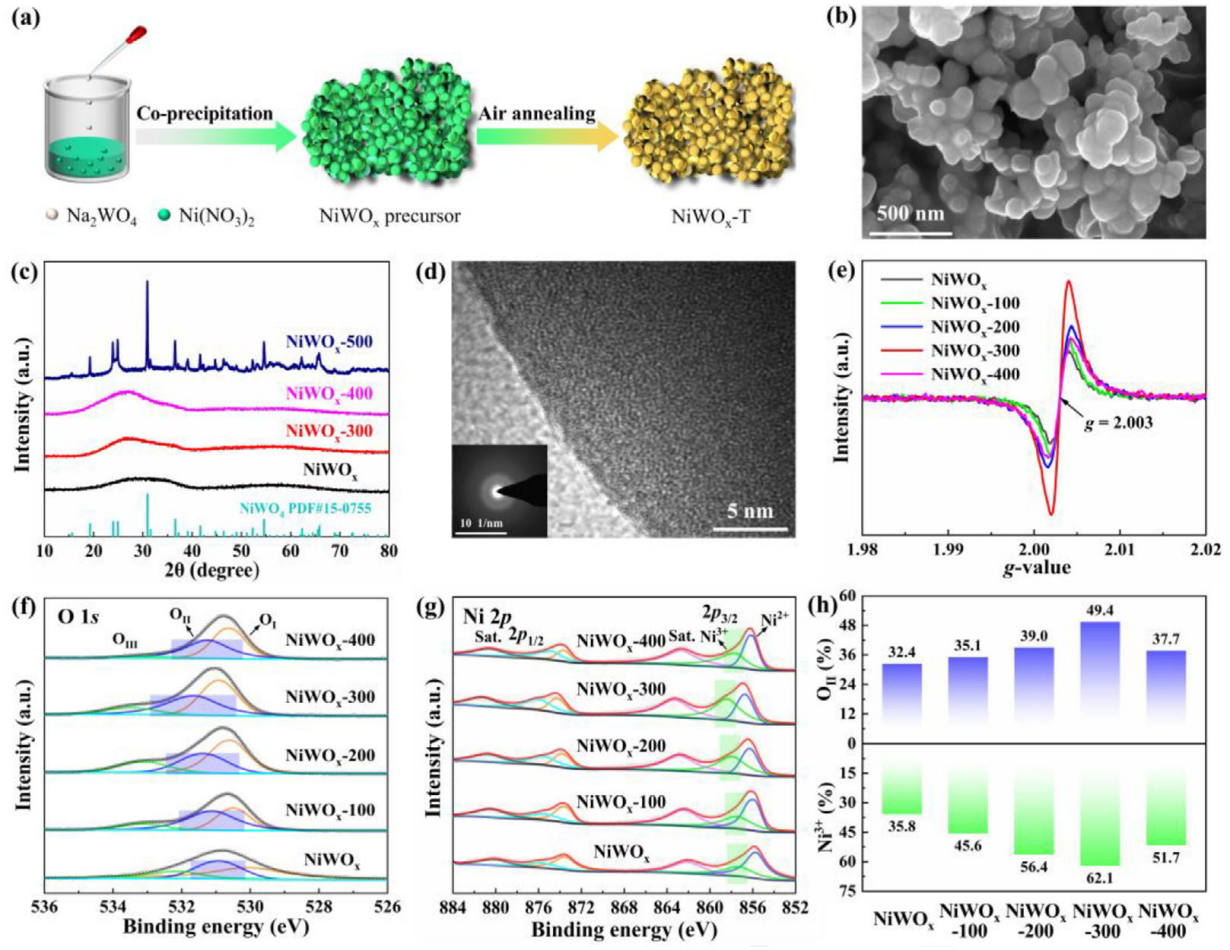
(a) Schematic diagram of the synthesis of NiWO_x_‐T catalysts. (b) SEM image of the NiWO_x_‐300. (c) XRD patterns of the NiWO_x_ precursor and partial NiWO_x_‐T. (d) HRTEM image with corresponding SAED (insertion) of the NiWO_x_‐300. (e) EPR spectra, deconvoluted (f) O 1*s* and (g) Ni 2*p* XPS spectra of the NiWO_x_ precursor and amorphous NiWO_x_‐T. (h) Comparison of O_II_ and Ni^3+^ composition in the NiWO_x_ precursor and amorphous NiWO_x_‐T.

Firstly, the NiWO_x_ precursor was synthesized via a one‐step co‐precipitation method. The precursor was subsequently annealed in air at temperatures ranging from 100 to 600 °C (details in Supporting Information), resulting in a series of final catalysts, denoted as NiWO_x_‐T (T: Annealing temperature) (Figure 1a). Scanning electron microscopy (SEM) and transmission electron microscopy (TEM) images show that the NiWO_x_‐T catalyst (exemplified by NiWO_x_‐300) consists of well‐defined spherical nanoparticles aggregated into clusters with homogeneous elemental distribution (Figures 1b and S1, S2). X‐ray diffraction (XRD) patterns reveal no distinct diffraction peaks for the NiWO_x_ precursor, NiWO_x_‐300, and NiWO_x_‐400 catalysts, indicating their amorphous characteristics (Figure 1c), which is supported by high‐resolution TEM (HRTEM) image and its selected area electron diffraction (SAED) pattern of NiWO_x_‐300 that no distinct diffraction spots or rings are observed (Figure 1d). The amorphous catalysts typically feature a highly defective structure and tolerance with abundant active sites, which could enhance their electrocatalytic performance. In contrast, the XRD pattern of NiWO_x_‐500 catalyst exhibits distinct diffraction peaks corresponding to the monoclinic NiWO_4_ phase (JCPDS No. 15‐0755) [31, 32], indicating the crystallization of NiWO_x_‐T catalysts after 500 °C. These findings confirm the amorphous nature of the NiWO_x_ catalyst with relatively low annealing temperatures.

The O_v_ in the NiWO_x_ precursor and amorphous NiWO_x_‐T catalysts were characterized by the unpaired electron trapped in the O_v_, which was studied by electron paramagnetic resonance (EPR). The EPR spectra are all isotropic with a *g* factor equal to 2.003, demonstrating the formation of O_v_ (Figure 1e) [33, 34, 35]. Higher EPR intensity indicates a greater concentration of unpaired electrons and O_v_. The results show that the amorphous NiWO_x_‐300 catalyst exhibits the highest intensity, followed by NiWO_x_‐200, NiWO_x_‐400, NiWO_x_‐100, and NiWO_x_ precursor, reflecting a corresponding trend in O_v_ concentration. The structure difference can also be suggested by the color difference of various NiWO_x_‐T catalysts (Figure S3) [36]. In addition, O_v_ was quantified by the x‐ray photoelectron spectroscopy (XPS) O 1*s* spectra for the NiWO_x_‐T catalysts (Figures 1f and S4–S7). The three peaks at around 530.5, 531.4, and 532.7 eV were attributed to the lattice oxygen (O_I_), oxygen vicinity of O_v_ (O_II_), and surface‐adsorbed water molecule (O_III_), respectively [26, 37, 38]. Particularly, the O_II_ composition of amorphous NiWO_x_‐300 catalyst obtained by integrating the peak area is up to 49.4% (Table S1), higher than those of NiWO_x_‐200 (39.0%), NiWO_x_‐400 (37.7%), NiWO_x_‐100 (35.1%), and NiWO_x_ precursor (32.4%), which is consistent with EPR analysis. Moreover, in the XPS Ni 2*p* spectra (Figure 1g), the Ni 2*p* 3/2 peaks for amorphous NiWO_x_‐T catalysts exhibit significant blue shifts compared to NiWO_x_ (Table S2), especially the subpeak for Ni^3+^ from deconvolution in NiWO_x_‐300 being 1.09 eV higher than that for the NiWO_x_ precursor. Besides, the composition of Ni^3+^ for amorphous NiWO_x_‐300 catalyst is 62.1%, higher than NiWO_x_‐200 (56.4%), NiWO_x_‐400 (51.7%), NiWO_x_‐100 (45.6%), and NiWO_x_ precursor (35.8%), which agrees with the order of O_II_ composition (Figure 1h). These findings indicate that NiWO_x_‐T with higher O_v_ concentration is more prone to electron loss, facilitating the generation of highly active Ni^3+^ species for the UOR. Furthermore, as indicated in Table S1, the proportion of the O_III_ component decreases with increasing annealing temperature, suggesting the gradual loss of surface‐adsorbed water. This dehydration process can promote the formation of O_v_ and helps maintain a highly local amorphous degree. However, excessive annealing induces structural reconstruction toward a more crystalline phase (Figure 1c), which in turn reduces the O_v_ concentration. The competition between these two opposing effects (O_v_ generation from dehydration and O_v_ elimination upon crystallization) results in a volcano‐type trend in O_v_ concentration with annealing temperature, peaking at 300 °C. This consistently explains the variation in O_v_ concentration across the annealed samples.

### 2.2 Electrochemical Evaluation and Kinetic Study Toward the UOR

**FIGURE 2:**
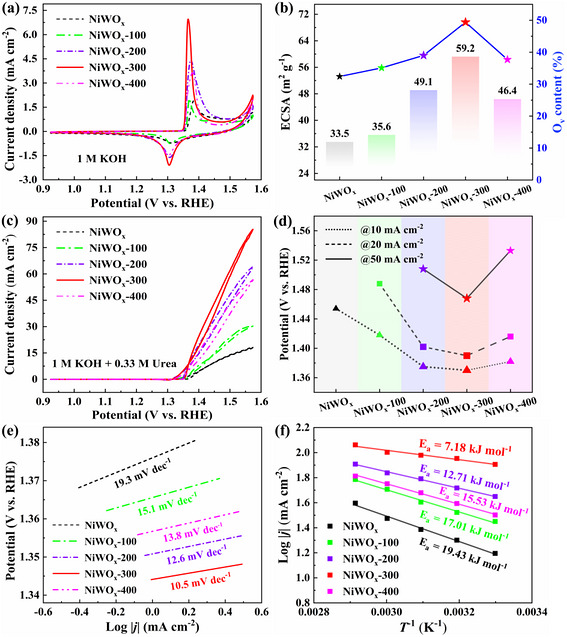
(a) CV curves of the NiWO_x_ precursor and amorphous NiWO_x_‐T in 1 M KOH electrolyte. (b) Correlation between the ECSA (bar) of the NiWO_x_ precursor and amorphous NiWO_x_‐T with O_v_ content (line + symbols). (c) CV curves of the NiWO_x_ precursor and amorphous NiWO_x_‐T in 1 M KOH with 0.33 M urea electrolyte. (d) Comparison of potentials of the NiWO_x_ precursor and amorphous NiWO_x_‐T for the UOR at current densities of 10, 20, and 50 mA cm^−2^. (e) Tafel plots and (f) Arrhenius plots for the UOR of the NiWO_x_ precursor and amorphous NiWO_x_‐T.

The capacity on NiOOH generation in amorphous NiWO_x_‐T catalysts and NiWO_x_ precursor were further accessed by cyclic voltammetry (CV) in a three‐electrode electrochemical cell with 1 M KOH (Figure 2a). A pair of redox peaks in the CV curves occurring prior to oxygen evolution reaction (OER) is attributed to the NiOOH redox couple [39, 40]. The electrochemical active surface area (ECSA) was determined by integrating the NiOOH reduction peak during the cathodic scan. Results indicate that the amorphous NiWO_x_‐300 catalyst exhibits the largest ECSA of 59.2 m^2^ g^−1^ (Figure 2b), followed by NiWO_x_‐200, NiWO_x_‐400, NiWO_x_‐100, and NiWO_x_ precursor, aligning with the trend in O_v_ concentration. This demonstrates that the increased O_v_ concentration in amorphous NiWO_x_ catalyst enhances NiOOH species generation. Subsequently, the UOR activities of amorphous NiWO_x_ catalysts were examined by CV in an electrolyte of 1 M KOH with 0.33 M urea (Figure 2c). It is obvious that the NiWO_x_‐300 catalyst exhibits the superior UOR activity achieving a peak current density of 85.4 mA cm^−2^, which is 4.7 times higher than that of NiWO_x_ precursor. The peak current density follows the trend: NiWO_x_‐300> NiWO_x_‐200> NiWO_x_‐400> NiWO_x_‐100> NiWO_x_ precursor, correlating with the previously observed O_v_ concentration. Notably, the peak mass activity of the NiWO_x_‐300 for the UOR reaches the ampere‐level (Figure S8). When normalized by ECSA, the specific activity of amorphous NiWO_x_ catalysts still increases consistently with higher O_v_ concentration (Table S3). Therefore, increasing O_v_ concentration in amorphous NiWO_x_ catalysts, not only facilitate NiOOH species generation under the UOR conditions, but also improves the intrinsic activity of active Ni species, delivering the excellent UOR performance. In addition, the peak current densities of the amorphous NiWO_x_ are significantly higher than those of crystalline catalysts (NiWO_x_‐500 and NiWO_x_‐600) (Figure S9), owing to the suitable binding energies compared to crystalline NiWO_4_ [41]. Besides, the applied potentials at current densities of 10, 20 and 50 mA cm^−2^ for all the catalysts are summarized in Figure 2d. Notably, the amorphous NiWO_x_‐300 catalyst requires only 1.37 and 1.39 V to achieve 10 and 20 mA cm^−2^, respectively, lower than NiWO_x_‐200, NiWO_x_‐400, NiWO_x_‐100, and NiWO_x_ precursor, showing consistent performance trends with O_v_ concentration. The potential differences are more significant at higher current density. The NiWO_x_‐300 catalyst only requires an applied potential of 1.47 V at 50 mA cm^−2^, reduced by 40 and 65 mV respectively compared to NiWO_x_‐200 and NiWO_x_‐400 catalysts, respectively. The enhanced electrocatalytic activity is attributed to the increased O_v_ content in amorphous NiWO_x_.

The reaction kinetics of amorphous NiWO_x_‐T catalysts were evaluated by Tafel slope and reaction activation energy (*E_a_*). Remarkably, the NiWO_x_‐300 exhibits the lowest Tafel slope of 10.5 mV dec^−1^, outperforming other NiWO_x_ catalysts (Figure 2e). The Tafel slopes follow the order: NiWO_x_‐300< NiWO_x_‐200< NiWO_x_‐400< NiWO_x_‐100< NiWO_x_ precursor. Because the fitted potential window encompasses both NiOOH formation and the UOR, this trend indicates that higher O_v_ concentrations improve the kinetics of both NiOOH generation and UOR. More precisely, after calculating the reaction activation energy by Arrhenius equation, the NiWO_x_‐300 catalyst displays the lowest *E_a_* of 7.18 kJ mol^−1^ compared to others (Figures 2f and S10). Similarly, the trend in *E_a_* from low to high aligns with that of the Tafel slope. Therefore, it can be noted that higher concentrations of O_v_ in amorphous NiWO_x_‐T lead to the lower activation energy required for the UOR.

### 2.3 Overall Urea Electrolysis for Energy‐Efficient Hydrogen Production

**FIGURE 3:**
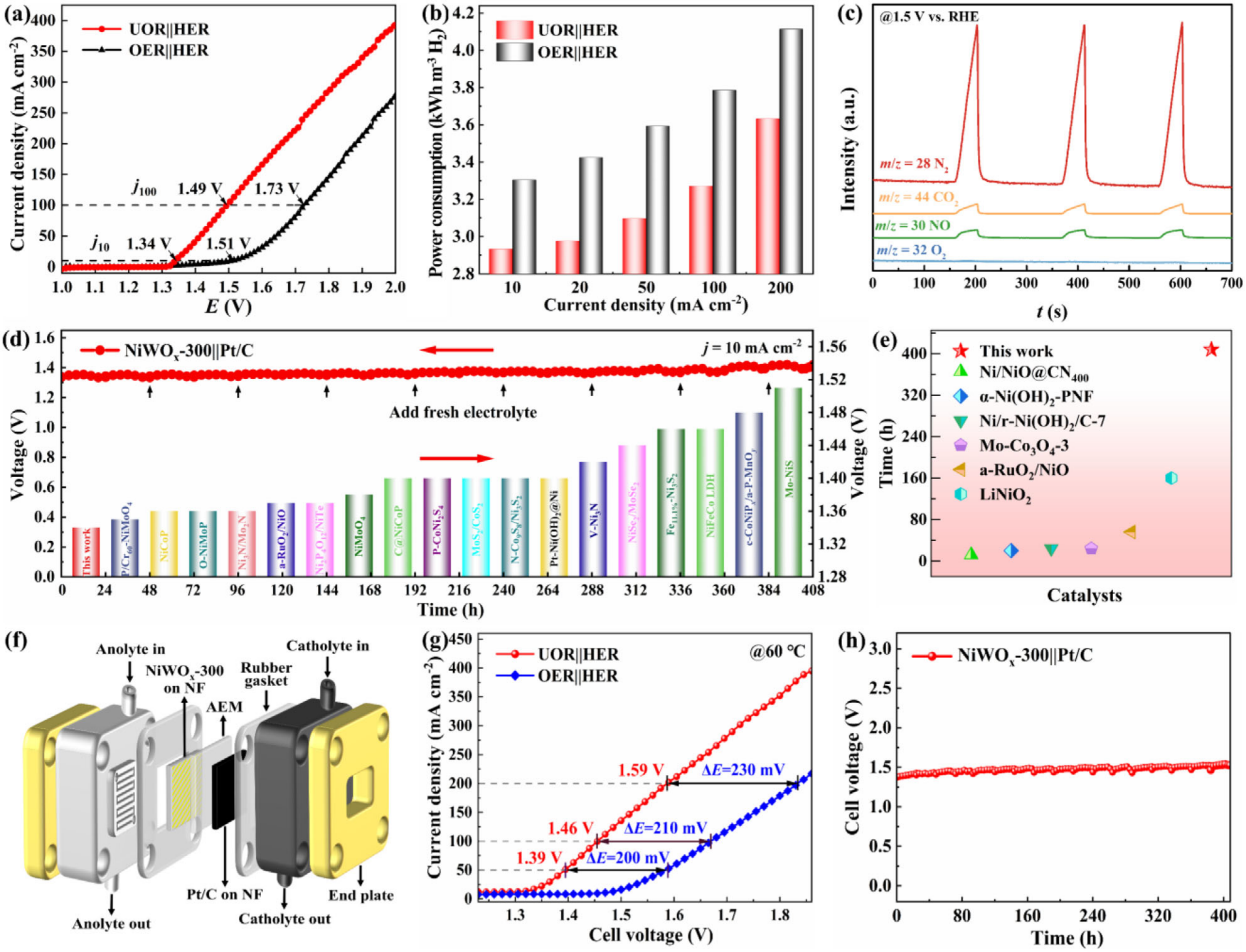
(a) Polarization curves and (b) power consumption of NiWO_x_‐300||Pt/C single‐cell electrolyzer for urea electrolysis and water splitting. (c) In situ DEMS for NiWO_x_‐300 at 1.5 V. (d) Stability of urea electrolysis at 10 mA cm^−2^ catalyzed by NiWO_x_‐300||Pt/C, and the activity comparison for NiWO_x_‐300||Pt/C with other studies. (e) Comparison of the stability of NiWO_x_‐300 with other O_v_‐rich catalysts. (f) Schematic diagram of AEMUE for urea electrolysis and water splitting for H_2_ production. (g) Polarization curves of AEMUE for urea electrolysis and water splitting at 60 °C. (h) Durability of AEMUE at 10 mA cm^−2^ for 408 h at room temperature in 1 M KOH with 0.33 M urea.

To develop the energy‐efficient hydrogen production system utilizing amorphous NiWO_x_‐T catalysts via the UOR compared to water splitting, a two‐electrode urea electrolyzer was assembled with NiWO_x_‐300 and a commercial Pt/C loaded on Ni foam (NF) as the anodic and cathodic catalysts, respectively. As shown in Figure 3a, urea electrolysis for hydrogen production exhibits a lower onset potential and a higher reaction rate compared to water splitting for hydrogen production, enabling the superior current density to be achieved at a lower voltage with consequently reduced energy consumption. Specifically, the cell voltages required to achieve 10 and 100 mA cm^−2^ for urea electrolysis are 1.34 and 1.49 V, respectively, considerably lower than the corresponding values for water splitting (1.51 and 1.73 V). The energy consumption for hydrogen production significantly decreases from 3.30 to 2.93 kWh m^−3^ H_2_ at 10 mA cm^−2^ (Figure 3b). This low energy consumption characteristic points out the potential of urea electrolysis for large‐scale industrial hydrogen production and energy conversion. Furthermore, the hydrogen production rate of urea electrolysis over NiWO_x_‐300 reaches 0.189 mmol h^−1^, which is more than four times higher than that of water splitting (Figure S11). A remarkable Faradaic efficiency (FE) of 98.2% toward hydrogen production was achieved for urea electrolysis (Figure S12), demonstrating exceptional charge utilization efficiency. Moreover, the product distribution at the anode was examined using in situ DEMS. After the background correction, the mass spectra at 1.5 V show a dominant signal at *m*/*z *= 28, along with two additional periodic signals at *m*/*z *= 30 and 44, corresponding to N_2_, NO, and CO_2_, respectively. Notably, no O_2_ signal (*m*/*z* = 32) was detected, indicating that pure UOR proceeds without detectable OER within 1.5 V (Figure 3c). The presence of a NO signal suggests that N‐containing species can undergo further oxidation beyond N_2_ under UOR conditions. Quantitative analysis of N‐containing products gave N‐selectivity of 73.5% for N_2_, 21.3% for NO_2_^−^, 5.2% for NO_3_^−^ at 1.5 V. (Figure S13).

Notably, the urea electrolysis for hydrogen production demonstrates outstanding stability, maintaining performance over 400 h at 10 mA cm^−2^ with minimal voltage fluctuation, outperforming most reported advanced catalysts (Figure 3d). This outstanding durability underscores the superior structural integrity and electrochemical stability of the NiWO_x_‐300 catalyst under prolonged operational conditions. The NiWO_x_‐300 electrode shows superior urea electrolysis activity as well as durability over other reported O_v_‐rich catalysts (Figure 3e, Table S4). Meanwhile, the catalyst can also stay a good long‐term stability under a higher current density (100 mA cm^−2^, Figure S14). The comprehensive performance highlights its promising application for energy‐efficient H_2_ production in urea electrolysis. Following the long‐term UOR test at a constant current density of 10 mA cm^−2^, XRD patterns confirm that the crystalline structure remains unchanged, indicating that the amorphous nature of NiWO_x_‐300 is preserved under long‐term operation (Figure S15). To examine the evolution of surface O_v_ during the test, the XPS O 1s spectra were deconvoluted. Quantitative analysis of O_I_, O_II_, and O_III_ components reveals a notable increase in the proportion of O_II_ (associated with oxygen vicinity of O_v_) from 34.3% to 43.2% after 100 h of UOR operation, indicating an increase in the surface O_v_ concentration (Figure S16a). This finding is further corroborated by the W 4*f* spectra, in which both the W 4*f*_7/2_ and W 4*f*_5/2_ peaks exhibit a distinct redshift after the stability test, consistent with a decrease in the oxidation state of W (Figure S16b). Collectively, these post‐test characterizations demonstrate that, the O_v_ concentration increases during prolonged UOR operation, which in turn reduces the valence state of W.

Furthermore, the performance of urea electrolysis for hydrogen production was evaluated in a customized flowing anion‐exchange membrane urea electrolyzer (AEMUE), featuring the NiWO_x_‐300@NF anode and the Pt/C@NF cathode directly contacting both sides of the AEMUE (Figure 3f). The polarization curves of the electrolyzer measured in 1 M KOH with and without 0.33 M urea are shown in Figure 3g. With the assistance of the AEMUE, urea electrolysis for hydrogen production achieves current densities of 50, 100, and 200 mA cm^−2^ at low cell voltages of only 1.39, 1.46, and 1.59 V, respectively, which are 200, 210, and 230 mV lower than those required for water splitting, demonstrating the energy‐saving advantage of urea electrolysis for hydrogen production in the AEMUE. In addition, the performance of urea electrolysis in AEMUE improved gradually with increasing operating temperatures (Figure S17a), attributed to enhanced reaction kinetics and ion diffusion at elevated temperatures [42, 43]. Notably, at 1.7 V, the current density increased from 158.7 mA cm^−2^ at 30 °C to 375.0 mA cm^−2^ at 80 °C (Figure S17b). Furthermore, Figure 3h presents the stability test of the NiWO_x_‐300||Pt/C AEMUE at a constant current density, during which only a slight voltage increase is observed over 400 h, illustrating its excellent long‐term durability. These findings demonstrate the promising potential for industrial applications in large‐scale energy‐efficient hydrogen production through urea electrolysis catalyzed by the NiWO_x_‐300 catalyst.

### 2.4 Catalytic Mechanism for the High UOR Performance

**FIGURE 4:**
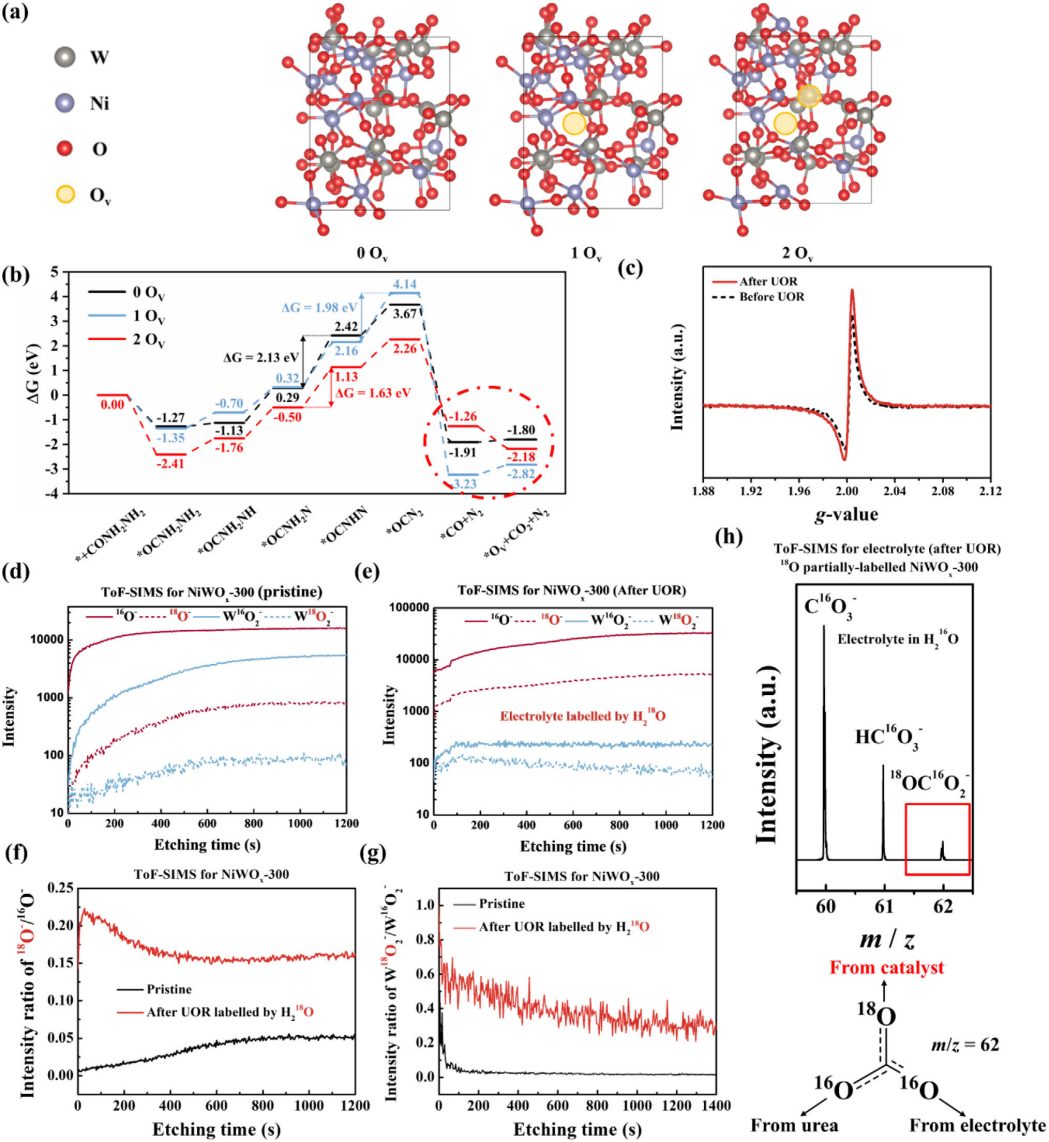
Demonstration of AOM. (a) The geometric structure of the amorphous NiWO_x_ with and without O_v_. (b) Gibbs free energy diagrams for the UOR on the amorphous NiWO_x_ with and without O_v_. (c) EPR spectra of NiWO_x_‐300 catalyst before and after the UOR. (d, e) Vertical distributions of O‐containing ToF‐SIMS ion fragments in NiWO_x_‐300 (d) before (pristine) and (e) after UOR in the electrolyte labelled by H_2_^18^O. (f, g) ToF‐SIMS intensity ratio of (f) ^18^O^−^/^16^O^−^ and (g) W^18^O_2_^−^/W^16^O_2_^−^ before (pristine, black curves) and after UOR (red curves) in the electrolyte labelled by H_2_^18^O. (h) ToF‐SIMS for the electrolyte after UOR with H_2_^16^O catalyzed by ^18^O partially‐labelled NiWO_x_‐300.

To elucidate the origin of the enhanced UOR activity and stability afforded by O_v_, DFT calculations were performed to investigate the reaction characteristics on amorphous NiWO_x_ catalysts. Figure 4a shows the idealized amorphous NiWO_x_ models, with 0, 1, and 2 O_v_ (denoted as 0 O_v_, 1 O_v_, and 2 O_v_, respectively) to examine the effects of increasing number of O_v_ on the UOR performance. As shown in Figure S18, in the absence of O_v_ (0 O_v_), urea is adsorbed at the Ni site in a parallel configuration by forming an O─Ni bond, and is stabilized by the surface O sites via hydrogen bonds. On the other hand, in the presence of O_v_ (1 O_v_ and 2 O_v_), as displayed in Figures S19 and S20, urea is adsorbed at the bridge site between W and Ni in a perpendicular configuration by forming Ni─O─W bonds, and is stabilized by the surface O sites via hydrogen bonds as well. As such, the bridge‐site adsorption of urea is generally stronger, especially with a higher degree of O_v_ (Figure S21). As the reaction proceeds, the dehydrogenated N atom in the adsorbed intermediate forms a bond with the surface O site bonded to the Ni site, constructing a Ni─O─C─N─O─Ni ring‐like configuration until N_2_ is released. As shown in Figure 4b, the adsorption strengths of these intermediates are similar between 0 O_v_ and 1 O_v_ systems, but are significantly stronger in the 2 O_v_ system. In addition, the potential‐determining step (PDS) is the oxidation of *OCNH_2_N to *OCNHN on 0 O_v_ and 2 O_v_ catalysts, and is that of *OCNHN to *OCN_2_ on 1 O_v_ catalyst. Compared to 0 O_v_, whose energy barrier (Δ*G*) for the PDS is 2.13 eV, creating surface O_v_ could effectively reduce the energy barrier for the PDS to 1.98 eV on 1 O_v_, and further to 1.63 eV on 2 O_v_. This demonstrates the trend of the superior UOR performance with the increasing number of O_v_ for amorphous NiWO_x_, which is consistent with the electrochemical test results.

After N_2_ is released, the *CO intermediate becomes strongly bonded to the surface metal (Ni and W) and/or O sites. On 0 O_v_, the C atom in *CO bonds to the O site; on 1 O_v_, the O atom in *CO bonds to the bridge site between Ni and W, while the C atom bonds to the O sites also in a bridge configuration; on 2 O_v_, the O atom in *CO bonds to the bridge site between Ni and W, while the C atom bonds to the O site. Remarkably, despite the different bonding modes among these three models, the *CO adsorption configurations thermodynamically prefer to undergo LOM‐like pathway, where surface O is activated to participate in the formation of CO_2_ rather than AEM‐like pathway, generating another O_v_ in the catalyst. Specifically, the formation of CO_2_ is exergonic (Δ*G* < 0) on 2 O_v_, while it is endergonic process (Δ*G* > 0) on 0 and 1 O_v_ (Figure S22), indicating a facile “amorphous oxygen” escape that increases O_v_ concentration during the UOR. Meanwhile, the energy difference between LOM‐like and AEM‐like pathways increases with O_v_ content (Figures S23–S25), suggesting that catalysts with higher O_v_ content preferentially undergo the LOM‐like pathway, further enhancing the O_v_ concentration. These DFT results are directly corroborated by increased EPR signal observed in NiWO_x_‐300 catalyst after the UOR, demonstrating a rise in O_v_ concentration (Figure 4c). These findings align with the gradual increase in current density under constant potential (Figure S26a) and maintained amorphous structure (Figure S26b), demonstrating that in situ generated O_v_ facilitates the UOR performance.

To further verify the participation of O from the amorphous NiWO_x_‐300 in UOR pathway, ex situ ToF‐SIMS with isotopic labelling was employed. H_2_^18^O was utilized to enrich the electrolyte (excluding urea) with ^18^O, while the O atoms in the pristine NiWO_x_‐300 remained ^16^O. Depth profiles of O‐containing ToF‐SIMS ion fragments (^16^O^−^, ^18^O^−^, W^16^O_2_^−^, W^18^O_2_^−^) obtained from the catalyst before (pristine) and after UOR operation in the ^18^O‐labelled electrolyte reveal a significant increase in ^18^O relative content within the catalyst after UOR (Figure 4d,e). Specifically, the intensity ratios of ^18^O^−^/^16^O^−^and W^18^O_2_^−^/W^16^O_2_^−^ on the surface increased from 0.0041 to 0.14 and from 0.28 to 1.0, respectively (Figure 4f,g). Meanwhile, depth profiles obtained from the catalyst after activation in ^18^O‐labelled 1 M KOH show similar trends (Figure S27). These indicate that O atoms from the catalyst participate in the UOR pathway, and ^18^O from the electrolyte subsequently exchanges into the catalyst, resulting in a ^18^O partially‐labelled NiWO_x_‐300. Furthermore, ^18^O partially‐labelled NiWO_x_‐300 was utilized to perform UOR in a normal H_2_^16^O electrolyte. The ToF‐SIMS result obtained from the dried electrolyte after UOR shows an obvious peak at *m*/*z* = 62, corresponding to a ^18^O‐labelled carbonate fragment ^18^OC^16^O_2_^−^ (Figure 4h). In the UOR pathway, the carbonate product contains three O atoms: One originates from urea (^16^O), one from the electrolyte (^16^O); and the other O originates from the catalyst (^18^O) confirmed by the detection of ^18^OC^16^O_2_^−^ (*m*/*z* = 62). This result provides direct evidence that, the oxygen from amorphous NiWO_x_‐300 is involved in the UOR pathway, consistent with a LOM‐like pathway. Besides, the result of in situ DEMS during UOR in H_2_^16^O catalyzed by ^18^O partially‐labelled NiWO_x_‐300 shows an obvious signal at *m*/*z* = 46, corresponding to ^18^OC^16^O (CO_2_ with ^16^O from urea and ^18^O from catalyst). This also supports the pathway involving oxygen from amorphous catalyst (Figure S28).

On this basis, we propose an “amorphous oxygen mechanism” (AOM), a sustainable catalytic pathway operative in amorphous oxide catalysts, which enables continuous regeneration and accumulation of O_v_ while preserving structural stability. Unlike the conventional AEM limited by PDS and LOM prone to oxygen loss and structural collapse, AOM leverages the uniquely disordered yet flexible atomic arrangement of amorphous catalysts to establish a self‐sustaining O_v_ cycle, maintaining a dynamic equilibrium between oxygen escape and vacancy stabilization. In general, the DFT results confirm the superior performance of O_v_‐rich amorphous NiWO_x_ for the electrocatalytic UOR by strengthening the intermediate adsorption to reduce energy barriers and by activating the surface oxygen to generate O_v_ in situ for further performance improvement.

## 3 Conclusion

In summary, this study systematically investigates the defective structure‐activity relationship concerning O_v_ concentrations in amorphous NiWO_x_‐T catalysts and their alkaline UOR activity. Physical and electrochemical characterizations reveal that, increased O_v_ content in amorphous NiWO_x_ enhances the formation of primary Ni^3+^ species and promotes the generation of highly active NiOOH under the UOR conditions. Furthermore, electrochemical characterization and theoretical calculations demonstrate that, the enrichment of O_v_ and the high content of NiOOH kinetically and thermodynamically facilitate the intrinsic UOR activity, leading to significantly improved peak current density and specific activity. Among these NiWO_x_‐T catalysts, NiWO_x_‐300 catalyst exhibits the superior UOR performance, achieving a low potential of 1.34 V at 10 mA cm^−2^ with a remarkable operational stability over 400 h. Moreover, it can be noted that the outstanding activity and energy‐saving property as well as the promising long‐term stability are achieved simultaneously in AEMUE for hydrogen production through urea electrolysis. Notably, theoretical calculations and relevant characterizations highlight the facile “amorphous oxygen” escaping mechanism that generates O_v_ in situ during the UOR, further enhancing the performance. The proposed “amorphous oxygen mechanism (AOM)” elucidates how the amorphous structure simultaneously enables robust activity and exceptional stability. This work, not only shines light on the systematic relationship between the O_v_ content and the alkaline UOR activity in amorphous Ni‐based catalysts, but also establishes a strategic framework for developing advanced electrocatalysts through defect engineering for energy‐saving hydrogen production.

## Author Contributions

**Yinuo Wang**: methodology, data curation, investigation, validation, resources, writing–review and editing, conceptualization, formal analysis, and writing–original draft. **Ke Zhang**: conceptualization, methodology, software, data curation, investigation, validation, formal analysis, resources, and writing–original draft. **Mingxing Zhou**: methodology, validation. **Shijin Zhang**: methodology, validation, and data curation. **Ling Zhao**: methodology, validation. **Gongjin Chen**: validation. **Luca Magagnin**: writing–review and editing, supervision. **Lu Wang**: conceptualization, methodology, investigation, validation, supervision, resources, project administration, funding acquisition, and writing–review and editing. **Yian Wang**: methodology, software, data curation, investigation, validation, formal analysis, visualization, writing–original draft, and resources. **Minhua Shao**: supervision, project administration, funding acquisition, writing–review and editing, and investigation.

## Conflicts of Interest

The authors declare no conflicts of interest.

## Supporting information




**Supporting File**: anie73098‐sup‐0001‐SuppMat.doc.

## Data Availability

The data that support the findings of this study are available from the corresponding author upon reasonable request.
