# Supplementary material for: Enriched and Sustained Oxygen Vacancies in Amorphous NiWOx Enhance and Stabilize Urea Electrooxidation
Source: Angew Chem Int Ed Engl. 2026 Jul 8;65(34):e2821661. doi: 10.1002/anie.2821661 (PMC13480780; doi:10.1002/anie.2821661)
Supplement: Supplementary file 1 [file ANIE-65-e2821661-s001.doc]

**Supporting Information**

**Enriched and Sustained Oxygen Vacancies in Amorphous NiWO_x_ Enhance and Stabilize Urea Electrooxidation**

Yinuo Wang,^[b]‡^ Ke Zhang,^[a]‡^ Mingxing Zhou,^[a]^ Shijin Zhang,^[a]^ Ling Zhao,^[a]^ Gongjin Chen,^[b]^ Luca Magagnin,^[c]^ Lu Wang,*^[a, b, c]^ Yian Wang,*^[d]^ Minhua Shao*^[b, e, f]^

^[a]^ School of Civil Engineering, Key Laboratory of Water Supply & Sewage Engineering of Ministry of Housing and Urban-rural Development, Chang’an University, Xi’an 710061, China

^[b]^ Department of Chemical and Biological Engineering, The Hong Kong University of Science and Technology, Clear Water Bay, Kowloon, Hong Kong 999077, China

^[c]^ Dipartimento di Chimica, Materiali e Ingegneria Chimica Giulio Natta, Politecnico di Milano, Via Edoardo Bassini 6, 20133 Milan, Italy

^[d]^ Dongguan Key Laboratory of Artificial Intelligence Design for Advanced Materials, School of Physical Sciences, Great Bay University, Dongguan 523000, China

^[e]^ CIAC-HKUST Joint Laboratory for Hydrogen Energy, Energy Institute, The Hong Kong University of Science and Technology, Clear Water Bay, Kowloon, Hong Kong 999077, China

^[f]^ Guangzhou Key Laboratory of Electrochemical Energy Storage Technologies, Fok Ying Tung Research Institute, The Hong Kong University of Science and Technology, Guangzhou 511458, China

^‡^ These authors contributed equally in this work.

*Corresponding authors:

Lu Wang (wangluxjtu@163.com)

Yian Wang (wya@gbu.edu.cn)

Minhua Shao (kemshao@ust.hk)

**Chemicals and materials**

Potassium hydroxide (KOH, GR, 95%) and urea (CO(NH_2_)_2_, AR, 99%) were purchased from Shanghai Aladdin Bio-Chem Technology Co., Ltd., China. Nickel(Ⅱ) nitrate hexahydrate (Ni(NO_3_)_2_·6H_2_O, AR, ≥ 98.0%) and sodium tungstate dihydrate (Na_2_WO_4_·2H_2_O, AR, ≥ 99.5%) were obtained from Sinopharm Chemical Reagent Co., Ltd., China. Isopropanol and ethanol were sourced from Tianjin Fuyu Fine Chemical Co., Ltd., China. The Ni foam (1.0 mm thick) and commercial Pt/C (50 wt.%, TEC10E50E) utilized in this study were procured from Kunshan Guangjiayuan New Material Co., Ltd., China and Tanaka Kikinzoku Kogyo (TKK), Japan, respectively. All chemicals were used as received without any further purification. Deionized water has been applied in all the experiments.

**Synthesis of NiWO_x_-T catalysts**

The NiWO_x_-T was prepared by a facile one-step co-precipitation method followed by calcination treatment. Firstly, the amorphous NiWO_x_ precursor was synthesized by a one-step co-precipitation method. In a typical synthesis, an aqueous solution of Na_2_WO_4_·2H_2_O (5 mmol in 30 mL H_2_O) was added dropwise into an aqueous solution of Ni(NO_3_)_2_·6H_2_O (5 mmol in 30 mL H_2_O) under vigorous stirring. After stirring for 2 h and aging for 12 h, the resulting precipitate was collected, washed thoroughly with deionized water, and dried at 70 °C to obtain the NiWO_x_ precursor. Subsequently, the NiWO_x_-T catalyst was successfully prepared by simply annealing the NiWO_x_ precursor, and the crystal structure and oxygen vacancy concentration of NiWO_x_-T were regulated by changing the annealing temperature. Specifically, the NiWO_x_ precursor was annealed at the target temperature T (T = 100 ℃, 200 ℃, 300 ℃, 400 ℃, 500 ℃ and 600 ℃) in a resistance furnace for 2 h with the temperature increase rate of 5 ℃ min^-1^. After cooled down to room temperature naturally, the resulting earthy-yellow powder was milled to obtain the NiWO_x_-T catalysts. The samples at different temperatures were labeled as NiWO_x_-T (T = 100, 200, 300, 400, 500, 600), respectively.

**Characterization**

The crystalline structures of the prepared catalysts were characterized by X-ray diffraction (XRD, SmartLab, Rigaku Corp., Japan) with Cu Kα radiation (40 kV, 30 mA) at a scan rate of 5 degree per minute. The surface microstructures and elemental composition of the samples were tested via field emission scanning electron microscopy (FESEM, MAIA3 LMH, TESCAN, Germany). Morphologies and nanostructures were further examined by transmission electron microscopy (TEM, JEM-2010F, JEOL, Japan). The surface elemental compositions and chemical states were examined via X-ray photoelectron spectroscopy (XPS, Kα, ThermoFisher Scientific, USA). The oxygen vacancies were characterized by electron paramagnetic resonance spectrometer (EPR, Bruker A300, Germany). The AOM was verified via ToF-SIMS (IONTOF M6) with a mass resolution of over 30000. A Bi ion source was utilized for the analysis beam at an energy of 30 keV. The negative mode was applied, with sputtering performed using Cs ions at an energy of 1 keV.

**Electrochemical measurements**

All electrochemical measurements were carried out using a CHI 660E electrochemical workstation (Shanghai Chenhua Instrument Co., Ltd., China). The urea electrooxidation performance of the as-prepared catalysts was evaluated using a standard three-electrode system with a carbon rod and a Hg/HgO electrode as counter and reference electrodes, respectively. The glassy carbon electrode (GCE) deposited by NiWO_x_-T catalysts was used as the working electrode. Specifically, 3 mg of prepared catalyst, 0.5 mL of isopropanol, 2 mL of deionized water, and 20 µL of 5 wt.% Nafion solution were sonicated for 30 min to obtain a homogeneous suspension. Then 5 µL of the suspension was dropped on polished GCE and dried overnight at room temperature.

The UOR activity of the NiWO_x_-T catalysts was evaluated by cyclic voltammetry (CV) and linear sweep voltammetry (LSV) using an electrochemical workstation (CHI 660E). The range of the CV and LSV curves was 0.924-1.574 V vs. RHE with a scan rate of 10 mV s^-1^. All potentials were converted to a reversible hydrogen electrode (RHE) by the following equation:

*E*_RHE_ = *E*_ref_ + 0.059 × pH + *E* _Hg/HgO_

where *E*_RHE_ is the potential *vs.* RHE, *E*_ref_ is the potential *vs.* Hg/HgO electrode, and *E* _Hg/HgO_ = 0.098 V vs. NHE at 25 ℃ (NHE: normal hydrogen electrode).

The Tafel plot was evaluated by fitting the linear part of the LSV data using the Tafel equation: *η* = *a* + *b* log(*j*)

where *η* is the overpotential, *a* is the intercept, *b* is the Tafel slope, and *j* is the current density.

**In situ DEMS measurements**

In situ DEMS (Shanghai Yuanfang Tech) was used for in situ detection of volatile gas products in UOR. A 1 M KOH + 0.33 M urea electrolyte was fed into the in situ DEMS specially designed reaction cell, and Ar was continuously passed into the electrolyte before and during the in situ DEMS measurements. The as-prepared catalyst ink was applied uniformly dropwise to hydrophobic carbon paper, treated as the working electrode after dried under an infrared light. A carbon rod and Ag/AgCl electrodes were served as the counter and reference electrodes, respectively. LSV was cyclically performed during in situ DEMS measurements, and the mass spectra were collected at 1.5 V.

**Electrochemical activation energy**

To investigate the temperature-dependent electrocatalytic behavior of NiWO_x_-T catalysts, LSV was conducted at different temperatures (ranging from 30 °C to 70 °C). The electrochemical activation energy (*E*_a_) of NiWO_x_-T catalysts can be calculated by the Arrhenius equation:^[1,2]^

where *j* is the current density, *T* is the absolute temperature, and *R* is the universal gas constant.

**Two-electrode urea electrolyzer**

The performance of hydrogen production through urea electrolysis was evaluated in a self-assembled two-electrode electrolyzer, with NiWO_x_-300 catalyst (2 mg) loaded nickel foam (NF: 0.5 cm × 0.5 cm) as anode (working electrode) and commercial Pt/C catalyst (2 mg) loaded NF as cathode (counter electrode). The polarized curves of water splitting and urea electrolysis for NiWO_x_-300||Pt/C system were tested using linear sweep voltammetry in 1 M KOH and 1 M KOH + 0.33 M urea electrolyte, respectively, with a cell voltage range of 1-2 V and a scan rate of 10 mV s^-1^. The power consumption of urea electrolysis for hydrogen production was calculated according to Faraday's law by the following equations:^[3]^

where *W* is the power consumption, *v* is the cell voltage, *n* is the electron number of HER, *F* is the Faraday constant, and *V_m_* is the molar volume of gas.

The hydrogen production rate of the NiWO_x_-300||Pt/C system was tested at a fixed cell voltage of 1.6 V in the H-type electrolyzer using amperometric current-time (i-t) curve technique. The Faraday efficiency toward hydrogen through urea electrolysis was calculated by the following equation:^[4]^

FE

where FE is the Faraday efficiency, *n* is the electron number of HER, *V* is volume of hydrogen collected from the cathode, *F* is the Faraday constant, *Q* is the charge through the electrode, and *V_m_* is the molar volume of gas.

**Anion exchange membrane urea electrolyzer (AEMUE)**

In this study, a flowing anion exchange membrane urea electrolyzer (AEMUE) was assembled. The anode and cathode were NiWO_x_-300 and Pt/C catalysts loaded on NF (1 cm × 1 cm), respectively, directly contacting on both sides of a piece of anion exchange membrane (Versogen, USA), pre-treated in 0.5 M KOH for 2 h prior to use. The anode and cathode compartments were filled with 1 M KOH with 0.33 M urea and pure 1 M KOH, respectively. Electrolyte was circulated using a peristaltic pump (BT100L+YT25, Leifu, China) at a flow rate of 10 mL min^-1^.

**Theoretical calculations**

All spin-polarized density functional theory (DFT) calculations were conducted via the Vienna Ab initio Simulation Package (VASP),^[5]^ utilizing the projector-augmented-wave (PAW) method^[6]^ and the Perdew-Burke-Ernzerhof (PBE) pseudopotentials.^[7]^ The cutoff energy for the plane-wave basis set was 400 eV. The convergence thresholds for energy and force were 10^-5^ eV and 0.02 eV/Å. The strong on-site Coulomb interaction between Ni 3*d* electrons was described by an effective *U* value of 7 eV via the DFT+U method.^[8]^ The dispersion corrections were calculated using the zero-damping DFT-D3 method of Grimme.^[9]^ The amorphous NiWO_x_ structure was obtained using the “melt-and quench” approach via DFT-based ab initio molecular dynamics (AIMD) simulation: after relaxation of the monoclinic NiWO_4_ crystalline structure, it was then melted and maintained at 5000 K in the NVT ensemble with Nosé-Hoover thermostat for 10 ps; the molten state was then quenched to 300 K at a rate of 300 K ps^-1^; the quenched state was then maintained at 300 K in the NVT ensemble for 10 ps. For the resulting amorphous structure, the (0 0 1) facet was used to construct the slab model owing to the lowest cleavage energy. ^[10]^ A vacuum layer of 15 Å and dipole correction were added along the z-direction to prevent periodic interaction of the slab. On the basis of this amorphous NiWO_x_ structure with no oxygen vacancies (denoted as 0 O_v_), 1 and 2 oxygen atoms were removed deliberately from the surface to form amorphous NiWO_x_ models with different amounts of oxygen vacancies (denoted as 1 O_v_ and 2 O_v_, respectively). A 2×2×1 Monkhorst-Pack grid was employed for sampling. The Gibbs free energy change (Δ*G*) was calculated via the computational hydrogen electrode (CHE) model: Δ*G* = Δ*E*_DFT_ + Δ*E*_ZPE_ – *T*Δ*S*,^[11]^ where *E*_DFT_ is the DFT-computed energy, *E*_ZPE_ is the zero-point energy, *T* is the temperature and *S* is the entropy. The correction to the Gibbs free energy was calculated by VASPKIT.^[12]^

**Supplementary Figures and Tables**

**Figure S1.** TEM image of the NiWO_x_-300 catalyst.

**Figure S2.** (a) SEM image of the NiWO_x_-300 catalyst and the corresponding energy dispersive spectroscopy of (b) Ni, (c) W and (d) O elements.

**Figure S3.** Digital photographs of the NiWO_x_ precursor and amorphous NiWO_x_-T.

**Figure S4.** The XPS (a) survey spectrum and high-resolution (b) Ni 2*p*, (c) W 4*f*, and (d) O 1*s* spectra of NiWO_x_-100 catalyst.

**Figure S5**. The XPS (a) survey spectrum and high-resolution (b) Ni 2*p*, (c) W 4*f*, and (d) O 1*s* spectra of NiWO_x_-200 catalyst.

**Figure S6.** The XPS (a) survey spectrum and high-resolution (b) Ni 2*p*, (c) W 4*f*, and (d) O 1*s* spectra of NiWO_x_-300 catalyst.

**Figure S7.** The XPS (a) survey spectrum and high-resolution (b) Ni 2*p*, (c) W 4*f*, and (d) O 1*s* spectra of NiWO_x_-400 catalyst.

**Figure S8.** CV curves (normalized by the catalyst mass) of the amorphous NiWO_x_-T and NiWO_x_ precursor in 1 M KOH with 0.33 M urea.

**Figure S9.** CV curves of the (a) NiWO_x_-500 and (b) NiWO_x_-600 catalysts in 1 M KOH with and without 0.33 M urea.

**Figure S10.** LSV curves of (a) NiWO_x_ precursor, (b) NiWO_x_-100, (c) NiWO_x_-200, (d) NiWO_x_-300, and (e) NiWO_x_-400 in 1 M KOH with 0.33 M urea at different temperatures.

**Figure S11**. H_2_ production rates of NiWO_x_-300||Pt/C H-type electrolyzer for urea electrolysis and water splitting.

**Figure S12.** Faradaic efficiency of NiWO_x_-300||Pt/C H-type electrolyzer for urea electrolysis.

**Figure S13.** (a) Product distribution of N-containing products at 1.5 V vs. RHE for 10 h. (b-d) Calibration curves of (b) NO_3_^-^, (c) NO_2_^-^ and (d) total N in liquid.

**Quantification of Nitrate (NO_3_⁻)**

NO_3_⁻ was quantified by measuring its absorbance at 220 nm using a UV Vis spectrophotometer (N4, INESA Analytical Instrument Co., Ltd). To correct for interference from dissolved organic matter, a second measurement was performed at 275 nm. Prior to analysis, 1.0 mL of 1 M hydrochloric acid and 0.1 mL of 0.8 % sulfamic acid solution were successively added to 50 mL of the sample. After thorough mixing, the absorbance at 220 nm and 275 nm was recorded. The corrected absorbance (A) was calculated as:

A = A_220_ – 2A_275_.

**Quantification of Nitrite (NO_2_⁻)**

NO_2_⁻ was quantified using the N‑(1‑naphthyl)‑ethylenediamine photometric method, which is based on the formation of a red azo dye with maximum absorbance at 540 nm. The chromogenic reagent was prepared by dissolving 20.0 g of sulfanilamide and 1.0 g of N‑(1‑naphthyl)‑ethylenediamine dihydrochloride in a mixture of 250 mL deionized water and 50 mL phosphoric acid, followed by dilution to 500 mL with deionized water. For analysis, 1.0 mL of this reagent was added to 50 mL of sample. After mixing and a 20‑min reaction, the absorbance at 540 nm was measured.

**Quantification of total N in liquid**

Total N in liquid was quantified by the alkaline potassium persulfate digestion, which oxidizes all N-containing species to NO_3_⁻ in liquid. The digestion reagent (alkaline potassium persulfate solution) was prepared by first dissolving 40.0 g of K_2_S_2_O_8_ in 600 mL of deionized water with heating at 50 °C. Separately, 15.0 g of NaOH was dissolved in 300 mL of deionized water. After cooling, the two solutions were combined and diluted to 1000 mL with deionized water.

For the digestion, a 10.0 mL aliquot of the sample was placed in a sealed colorimetric tube, followed by the addition of 5.0 mL of the alkaline persulfate solution. The mixture was heated in an autoclave at 120 °C for 40 min. After cooling, 1.0 mL of diluted hydrochloric acid (1:9, v/v) was added, and the solution was diluted to 25 mL with deionized water. The absorbance was measured at 220 nm and 275 nm, and the corrected absorbance was obtained using A = A_220_ – 2A_275_. The total nitrogen concentration was determined from a pre‑established calibration curve.

**Quantification of N_2_**

Since the gaseous N-containing products of UOR are basically composed of N_2_, quantification of N_2_ is obtained by subtracting the total N in liquid after the reaction from the total N in liquid before the reaction.

**Figure S14.** Long-term stability of NiWO_x_-300 at 100 mA cm^-2^ for 144 h at room temperature in 1 M KOH with 0.33 M urea.

**Figure S15.** XRD patterns of NiWO_x_-300/NF before and after 100-h UOR test.

**Figure S16.** (a) Deconvoluted O 1*s* and (b) W 4*f* XPS spectra of NiWO_x_-300/NF before and after 100-h UOR test.

An additional peak (O_ⅠV_) appears at 535.6 eV, which is attributed to Nafion binder.^[13]^

Quantitative analysis of the O 1*s* spectra reveals that before UOR test, the proportions of O_Ⅰ_ (lattice oxygen), O_ⅠⅠ_ (oxygen vicinity of O_v_), and O_ⅠⅠⅠ_ (surface-adsorbed water species) are 40.5%, 34.3% and 25.2%, respectively. After 100h of UOR test, these proportions shift to 43.4%, 43.2% and 13.3%, respectively.

**Figure S17.** (a) Polarization curves of AEMUE for urea electrolysis at different temperatures. (b) Performance comparison of AEMUE for urea electrolysis at different temperatures at a cell voltage of 1.7 V.

**Figure S18.** Optimized configurations of UOR intermediates on 0 O_v_.

**Figure S19.** Optimized configurations of UOR intermediates on 1 O_v_.

**Figure S20.** Optimized configurations of UOR intermediates on 2 O_v_.

**Figure S21.** Free energy barriers of the amorphous NiWO_x_ with and without O_v_ for CO(NH_2_)_2_ adsorption.

**Figure S22**. Free energy barriers of the amorphous NiWO_x_ with and without O_v_ for CO_2_ desorption.

**Figure S23.** Gibbs free energy diagrams for LOM-like and AEM-like UOR on 0 O_v_.

**Figure S24.** Gibbs free energy diagrams for LOM-like and AEM-like UOR on 1 O_v_.

**Figure S25.** Gibbs free energy diagrams for LOM-like and AEM-like UOR on 2 O_v_.

**Figure S26.** (a) Amperometric i-t curve of NiWO_x_-300/NF electrode at 1.574 V *vs.* RHE in 1 M KOH with 0.33 M urea. (b) XRD pattern of the NiWO_x_-300 after UOR.

**Figure S27**. (a, b) Vertical distributions of O-containing ToF-SIMS ion fragments in NiWO_x_-300 (a) before (pristine, same with Figure 4d) and (b) after activation in ^18^O-labeled 1 M KOH. (c, d) ToF-SIMS intensity ratio of (c) ^18^O^-^/^16^O^-^ and (d) W^18^O_2_^-^/W^16^O_2_^-^ before (pristine, black curves) and after activation (red curves) in ^18^O-labeled 1 M KOH.

**Figure S28**. In situ DEMS during UOR in H_2_^16^O catalyzed by ^18^O partially-labelled NiWO_x_-300 at 1.5 V.

**Table S1.** Comparison of O 1*s* XPS results for amorphous NiWO_x_-T catalysts and NiWO_x_ precursor.

| Catalysts | O_Ⅰ_ | |  | O_Ⅱ_ | |  | O_Ⅲ_ | |
| --- | --- | --- | --- | --- | --- | --- | --- | --- |
|  | BE  (eV) | Composition  (%) |  | BE  (eV) | Composition  (%) |  | BE  (eV) | Composition  (%) |
| NiWO_x_ | 530.09 | 48.2 |  | 531.03 | 32.4 |  | 532.18 | 19.4 |
| NiWO_x_-100 | 530.37 | 46.1 |  | 531.17 | 35.1 |  | 532.94 | 18.8 |
| NiWO_x_-200 | 530.80 | 45.7 |  | 531.42 | 39.0 |  | 533.01 | 15.3 |
| NiWO_x_-300 | 531.11 | 39.9 |  | 531.82 | 49.4 |  | 533.67 | 10.7 |
| NiWO_x_-400 | 530.60 | 53.6 |  | 531.25 | 37.7 |  | 532.97 | 8.7 |

where O_Ⅰ_ is the lattice oxygen, O_Ⅱ_ is the oxygen vicinity of O_v_, O_Ⅲ_ is the surface adsorbed water molecule, and BE is the binding energy of the O 1*s* peaks.

**Table S2.** Comparison of Ni 2*p* XPS results for amorphous NiWO_x_-T catalysts and NiWO_x_ precursor.

| Catalysts | Ni^3+^ | |  | | Ni^2+^ | |
| --- | --- | --- | --- | --- | --- | --- |
|  | BE  (eV) | Composition  (%) |  | BE  (eV) | | Composition  (%) |
| NiWO_x_ | 857.30 | 35.8 |  | 855.74 | | 64.2 |
| NiWO_x_-100 | 857.46 | 45.6 |  | 855.91 | | 54.4 |
| NiWO_x_-200 | 857.96 | 56.4 |  | 856.29 | | 43.6 |
| NiWO_x_-300 | 858.39 | 62.1 |  | 856.72 | | 37.9 |
| NiWO_x_-400 | 857.63 | 51.7 |  | 856.13 | | 48.3 |

where BE is the binding energy of the Ni 2*p* peaks.

**Table S3.** Electrochemical activities of NiWO_x_ precursor and amorphous NiWO_x_-T catalysts.

| Catalysts | ECSA  (m^2^ g^-1^) | Mass activity  (mA mg^-1^) | Specific activity  (A m^-2^) |
| --- | --- | --- | --- |
| NiWO_x_  NiWO_x_-100  NiWO_x_-200  NiWO_x_-300  NiWO_x_-400 | 33.5  35.6  49.1  59.2  46.4 | 215.7  370.1  761.0  1014.2  672.0 | 6.4  10.4  15.5  17.1  14.5 |

The specific activity is defined as the ratio of peak mass activity to ECSA. The ECSA can be calculated using the equation: ECSA = *Q*/(*m*×*q*), where *Q* is the total charge for the reduction of NiOOH to Ni(OH)_2_, *m* is the mass of the catalyst, and *q* is the charge associated with the formation of a monolayer of Ni(OH)_2_ (0.257 mC cm^-2^).^[14,15]^

**Table S4.** Comparison of the performance of hydrogen production through urea electrolysis of NiWO_x_-T with other studies.

| Catalyst | Electrolyte  (KOH + urea) | Voltage  (*E* V @ *j* mA cm^-2^) | Time  (*T* h @ *j* mA cm^-2^) | Reference |
| --- | --- | --- | --- | --- |
| **NiWO_x_-300** | **1.0 M + 0.33 M** | **1.34@10**  **1.49@100** | **408@10** | **This work** |
| Pt-Ni(OH)_2_@Ni | 1.0 M + 0.33 M | 1.40@10 | 36@~20 | 3 |
| Ni_3_N/Mo_2_N | 1.0 M + 0.33 M | 1.36@10 | 50@10 | 16 |
| V-Ni_3_N | 1.0 M + 0.50 M | ~1.42@10 | 200@10 | 17 |
| O-NiMoP | 1.0 M + 0.50 M | 1.36@10 | 10@20 | 18 |
| Fe_11.1%_-Ni_3_S_2_ | 1.0 M + 0.33 M | 1.46@10 | 20@10 | 19 |
| NiMoO_4_ | 1.0 M + 0.50 M | 1.38@10 | 50@100 | 20 |
| NiCoP | 1.0 M + 0.50 M | 1.36@10 | 40@10 | 21 |
| a-RuO_2_/NiO | 1.0 M + 0.33 M | 1.37@10 | 56@10 | 22 |
| NiSe_2_/MoSe_2_ | 1.0 M + 0.50 M | 1.44@10 | 48@10 | 23 |
| P-CoNi_2_S_4_ | 1.0 M + 0.50 M | 1.40@10 | 100@10 | 24 |
| N-Co_9_S_8_/Ni_3_S_2_ | 1.0 M + 0.50 M | 1.40@10 | 20@20 | 25 |
| MoS_2_/CoS_2_ | 1.0 M + 0.50 M | 1.40@10 | 50@~50 | 26 |
| c-CoNiP*_x_*/a-P-MnO*_y_* | 1.0 M + 0.50 M | ~1.48@10 | 40@50 | 27 |
| P/Cr_60_-NiMoO_4_ | 1.0 M + 0.33 M | 1.35@10 | 60@100 | 28 |
| Mo-NiS | 1.0 M + 0.50 M | 1.51@10 | 12@10 | 29 |
| NiFeCo LDH | 1.0 M + 0.33 M | 1.46@10 | 10@10 | 30 |
| Ni_2_P_4_O_12_/NiTe | 1.0 M + 0.33 M  1.0 M + 0.50 M | 1.37@10  1.34@10 | 500@100 | 31 |
| C@NiCoP | 1.0 M + 1.0 M | 1.40@10 | 36@~150 | 32 |
| Ni/NiO@CN_400_ | 1.0 M + 0.33 M | 1.50@10 | 20@~10 | 33 |
| LiNiO_2_ | 1.0 M + 0.50 M | - | 160@10 | 34 |
| Ni/r-Ni(OH)_2_/C-7 | 1.0 M + 0.33 M | 1.45@10 | 24@~10 | 35 |
| α-Ni(OH)_2_-PNF | 1.0 M + 0.33 M | - | 20@~10 | 36 |
| Mo-Co_3_O_4_-3 | 1.0 M + 0.33 M | 1.42@10 | 24@10 | 37 |

where *E* is the electrolysis voltage, *j* is the current density, and *T* is the electrolysis time.

**References:**

[1]	Y. Zhang, Y. Lei, Y. Yan, W. Cai, J. Huang, Y. Lai, Z. Lin, *Appl. Catal. B: Environ. Energy* **2024**, *353*, 124064.

[2]	M. M. Meshesha, D. Chanda, B. L. Yang, *Appl. Catal. B: Environ. Energy* **2024**, *344*, 123635.

[3]	M. Zhong, M. Xu, S. Ren, W. Li, C. Wang, M. Gao, X. Lu, *Energy Environ. Sci.* **2024**, *17*, 1984-1996.

[4]	G. Zhan, L. Hu, H. Li, J. Dai, L. Zhao, Q. Zheng, X. Zou, Y. Shi, J. Wang, W. Hou, Y. Yao, L. Zhang, *Nat. Commun.* **2024**, *15*, 5918.

[5]	G. Kresse, J. Furthmüller, *Phys. Rev. B* **1996**, *54*, 11169-11186.

[6]	P. E. Blöchl, *Phys. Rev. B* **1994**, *50*, 17953-17979.

[7]	J. P. Perdew, K. Burke, M. Ernzerhof, *Phys. Rev. Lett.* **1996**, *77*, 3865-3868.

[8]	S. L. Dudarev, G. A. Botton, S. Y. Savrasov, C. J. Humphreys, A. P. Sutton, *Phys. Rev. B* **1998**, *57*, 1505-1509.

[9]	S. Grimme, J. Antony, S. Ehrlich, H. Krieg, *J. Chem. Phys.* **2010**, *132*, 154104.

[10]	L. Wang, S. Zhu, Y. Wang, Z. Liu, Y. Liu, Q. Wang, M. Gu, K. Li, X. Sun, L. Yang, M. Shao, *Chem. Eng. J.* **2023**, *460*, 141826.

[11]	J. K. Nørskov, J. Rossmeisl, A. Logadottir, L. Lindqvist, J. R. Kitchin, T. Bligaard, H. Jónsson, *J. Phys. Chem. B* **2004**, *108*, 17886-17892.

[12]	V. Wang, N. Xu, J.-C. Liu, G. Tang, W.-T. Geng, *Comput. Phys. Commun.* **2021**, *267*, 108033.

[13]	A. K. Friedman, W. Shi, Y. Losovyj, A. R. Siedle, L. A. Baker, Journal of The Electrochemical Society 2018, 165, H733.

[14]	L. Wang, T. Du, J. Cheng, X. Xie, B. Yang, M. Li, *J. Power Sources* **2015**, *280*, 550-554.

[15]	G. Das, R. M. Tesfaye, Y. Won, H. H. Yoon, *Electrochim. Acta* **2017**, *237*, 171-176.

[16]	T. Wang, L. Miao, S. Zheng, H. Qin, X. Cao, L. Yang, L. Jiao, *ACS Catal.* **2023**, *13*, 4091-4100.

[17]	R.-Q. Li, Q. Liu, Y. Zhou, M. Lu, J. Hou, K. Qu, Y. Zhu, O. Fontaine, *J. Mater. Chem. A* **2021**, *9*, 4159-4166.

[18]	H. Jiang, M. Sun, S. Wu, B. Huang, C.-S. Lee, W. Zhang, *Adv. Funct. Mater.* **2021**, *31*, 2104951.

[19]	W. Zhu, Z. Yue, W. Zhang, N. Hu, Z. Luo, M. Ren, Z. Xu, Z. Wei, Y. Suo, J. Wang, *J. Mater. Chem. A* **2018**, *6*, 4346-4353.

[20]	Z.-Y. Yu, C.-C. Lang, M.-R. Gao, Y. Chen, Q.-Q. Fu, Y. Duan, S.-H. Yu, *Energy Environ. Sci.* **2018**, *11*, 1890-1897.

[21]	X. Ding, L. Pei, Y. Huang, D. Chen, Z. Xie, *Small* **2022**, *18*, 2205547.

[22]	L. Li, X. Zhang, M. Humayun, X. Xu, Z. Shang, Z. Li, M. F. Yuen, C. Hong, Z. Chen, J. Zeng, M. Bououdina, K. Temst, X. Wang, C. Wang, *ACS Nano* **2024**, *18*, 1214-1225.

[23]	X. Xu, H. Liao, L. Huang, S. Chen, R. Wang, S. Wu, Y. Wu, Z. Sun, H. Huang, *Appl. Catal. B: Environ.* **2024**, *341*, 123312.

[24]	X. F. Lu, S. L. Zhang, W. L. Sim, S. Gao, X. W. Lou, *Angew. Chem. Int. Ed.* **2021**, *60*, 22885-22891.

[25]	H. Xie, Y. Feng, X. He, Y. Zhu, Z. Li, H. Liu, S. Zeng, Q. Qian, G. Zhang, *Small* **2023**, *19*, 2207425.

[26]	T. L. Luyen Doan, D. C. Nguyen, K. Kang, A. Ponnusamy, H. I. Eya, N. Y. Dzade, C. S. Kim, C. H. Park, *Appl. Catal. B: Environ.* **2024**, *342*, 123295.

[27]	L. Qiao, A. Zhu, D. Liu, J. Feng, Y. Chen, M. Chen, P. Zhou, L. Yin, R. Wu, K. W. Ng, H. Pan, *Chem. Eng. J.* **2023**, *454*, 140380.

[28]	Y. Li, H. Guo, Y. Zhang, R. Song, *Appl. Catal. B: Environ.* **2024**, *341*, 123296.

[29]	Y. Zhou, Y. Wang, D. Kong, Q. Zhao, L. Zhao, J. Zhang, X. Chen, Y. Li, Y. Xu, C. Meng, *Adv. Funct. Mater.* **2023**, *33*, 2210656.

[30]	C. Fang, D. Zhang, X. Wang, R. Li, *Inorg. Chem. Front.* **2022**, *9*, 3643-3653.

[31]	P. Guo, S. Cao, W. Huang, X. Lu, W. Chen, Y. Zhang, Y. Wang, X. Xin, R. Zou, S. Liu, X. Li, *Adv. Mater.* **2024**, *36*, 2311766.

[32]	M. Shi, F. Sultana, X. Qin, P. Zhang, K. Qian, T. Wei, Y. Duan, T. Li, J. Bai, R. Li, *Appl. Catal. B: Environ. Energy* **2025**, *371*, 125210.

[33]	X. Ji, Y. Zhang, Z. Ma, Y. Qiu, *ChemSusChem* **2020**, *13*, 5004-5014.

[34]	W.-K. Han, J.-X. Wei, K. Xiao, T. Ouyang, X. Peng, S. Zhao, Z.-Q. Liu, *Angew. Chem. Int. Ed.* **2022**, *61*, e202206050.

[35]	C. Gao, G. Wei, C. Wang, X. Zhou, X. Zhao, Q. Zhao, S. Wang, F. Kong, *J. Alloy. Compd.* **2023**, *959*, 170545.

[36]	Y. Li, F. Luo, Y. Xie, C. Chang, M. Xie, Z. Yang, *Int. J. Hydrogen Energy* **2023**, *48*, 9155-9162.

[37]	P. Mannu, R.K. Dharman, T.T.T. Nga, A. Mariappan, Y.-C. Shao, H. Ishii, Y.-C. Huang, A. Kandasami, T.H. Oh, W.-C. Chou, C.-L. Chen, J.-L. Chen, C.-L. Dong, *Small* **2025,** *21*, 2403744.
